# The potential of psychiatric outpatient centers to reduce the length of stay in inpatient facilities and the negative impact of COVID-19 on the availability of psychiatric services: the case of Latvia

**DOI:** 10.3389/frhs.2024.1348919

**Published:** 2024-07-04

**Authors:** Maris Taube

**Affiliations:** ^1^ Department of Psychosomatic Medicine and Psychotherapy, Riga Stradiņš University, Riga, Latvia; ^2^ Department for Depression and Crisis, Riga Center of Psychiatry and Narcology, Riga, Latvia

**Keywords:** hospital beds, number of patients, psychiatry, outpatient care, length of stay, COVID-19

## Abstract

The move from psychiatric hospitals to community-based care is the goal of policies in many countries. Latvia has attempted to reach this goal by establishing two outpatient centers in Riga. Since 2005, when the first outpatient centers opened, the ability of day clinics to reduce the total length of stay for hospital inpatients has been observed, although using the outpatient centers did not affect the number of patients treated. The open-door inpatient wards of the centers also attracted a new patient group. However, due to the COVID-19 pandemic, the number and length of stay of both outpatients and inpatients at psychiatric hospitals decreased. Therefore, other factors that can affect the move from psychiatric hospital inpatient care to outpatient centers should be further investigated.

## Introduction

The goal for developing psychiatric services in Europe and globally entails treating more psychiatric patients outside of inpatient facilities. The objective is to shorten and reduce the frequency of hospital treatments while preventing psychotic patients from engaging in behavior that could result in the involvement of the judicial psychiatric system (1). However, this change is not simple to implement, as the availability and types of outpatient services are as important as the quality of the services and their measurements (2). Currently, the balanced care model, which includes both hospital and community care (3), is the most widely accepted approach (4). However, different approaches to developing outpatient treatment are offered in countries with different income levels. In low-income countries, the strongest emphasis should be placed on the involvement of family doctors, while in middle- and high-income countries (and in addition to the involvement of primary care doctors) outpatient/ambulatory clinics, community mental health teams, community-based residential facilities, specialized outpatient and ambulatory clinics, acute day hospitals, occupation, rehabilitation, and other services should also be developed. As the psychiatric care systems of each country differ, countries often choose their own path to achieve a balanced care goal (5). Some of the most common mistakes when developing services outside of traditional psychiatric hospitals include the hasty closures of inpatient psychiatric beds without creating community-based services, insufficient stakeholder involvement, and the implementation of reforms due to the ideological or political interests of politicians in power at the time (6).

## Policy options and implications

Latvia is a country in the European Union (EU) with a high income level and a population of 1,883,379 people (7). It has 122 psychiatric beds per 100,000 inhabitants, which places it among the countries with one of the highest rates of psychiatric beds in the EU (8). To reduce the number of hospital inpatients and length of hospital stays, Latvia established two outpatient centers in Riga, the capital city, covering a care area of approximately 700,000 inhabitants. The development of outpatient centers in Riga began gradually in 2005 with the opening of the first outpatient center, followed by the opening of the second center in 2013. A third center is planned to be operational in 2024. The centers provide outpatient psychiatric appointments and day admissions, with services that are run by various psychiatric rehabilitation team specialists (visual arts, music, drama, dance and movement therapy, physiotherapy, psychotherapy, and occupational therapy). There is also an open-door inpatient ward in each center, which has reduced the equivalent number of beds in traditional psychiatric hospitals in Riga. These wards are meant to treat most patients with therapeutic-resistant depression, severe anxiety conditions, and eating disorders, including patients with psychotic disorders who want to receive treatment and are able to follow an open-type unit regimen. These departments provide diagnostic, medical, and non-medical treatments (such as psychotherapy, art, and music therapy) as well as rehabilitation in a free environment without any restrictive measures. Patients are free to move outside the department, but they must also follow the agreed treatment and rehabilitation plan, which includes taking medicines and participating in the treatment sessions. The two outpatient centers were established in two city districts far from the primary psychiatric hospital established in 1824. This was done to ensure that the new centers would be closer to the patients’ places of residence, would not be associated with institutional treatment in psychiatric hospitals, and would offer a more comprehensive range of services (9). The two new outpatient centers with open-door inpatient wards have a capacity of approximately 18,000–20,000 outpatient psychiatric appointments annually, approximately 250–300 outpatient admissions, and treat approximately 310–360 inpatients at each center. The mean length of treatment in the day hospitals is 20 days, but in the inpatient wards it is 30 days.

**Figure 1:**
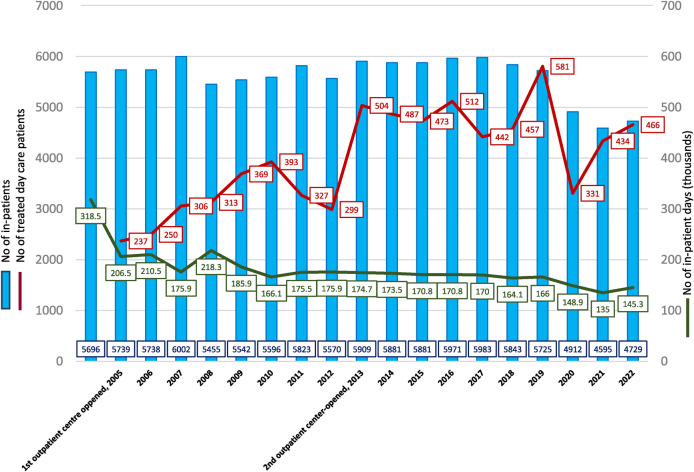
Number of inpatients, number of outpatients, and number of inpatient days, 2004–2022 [blue—number of inpatients, red—number of treated outpatients, green—number of inpatient days (thousands)].

The data in Figure 1 show that, upon its opening in 2005, the first outpatient center contributed to a reduction in the length of stay for psychiatric hospital inpatients. The duration of hospital stays decreased as the number of day patients in the outpatient centers increased. With the opening of the second outpatient center in 2013, the number of day patients increased by approximately 40%. However, the total duration of inpatient hospital stays remained at approximately 160,000–170,000 bed-days until 2020. It is possible that the day clinics contributed to a reduction in the duration of hospital stays as patients were admitted to the hospital only when the clinic's options were exhausted. Alternatively, the clinic would also replace inpatient treatment after a patient's condition was stabilized and intensive therapy in a closed hospital was no longer needed.

In contrast, the number of hospital-treated patients remained the same, irrespective of how the day clinics were run. The transfer of patients from inpatient wards to the newly opened outpatient centers and their open-door wards did not affect the total number of hospital-treated patients. This may be because more patients diagnosed with depression—who previously did not have access to treatment or avoided receiving it in the closed wards of the psychiatric hospital—were treated in the new open-door wards (see Table 1). For example, in 2019, of the patients treated in the two open-door wards of the outpatient centers, approximately 40% had mood (affective) disorders. In contrast, only 12.9% of the patients treated in the psychiatric hospital had mood disorders. If we compare the percentage of patients by mental health diagnoses at the psychiatric hospital before (2004) and after (2019) the opening of two new centers (see Table 2), the proportion of mood (affective) disorders increased much slower—from 6.8% to 12.9% and did not reach 40%. Neurotic, stress-related, and somatoform disorders, disorders of adult personality and behavior, and even schizophrenia, schizotypal, and delusional disorders decreased during that period (see Table 2). Hence, while the open-door inpatient wards expanded the range of services that were offered at the psychiatric hospital, they did not improve treatment options for patients with psychotic, organic psychiatric disorders as much as expected. This is especially evident when compared to the treatment options available for patients with depressive disorders. Even though more patients with depression were treated in the new open-door inpatient wards, the number of hospitalized patients with disorders, such as psychotic and/or organic psychiatric disorders, appeared to decrease. The reasons for this are not entirely clear and require further investigation.

**Table 1:** Percentage of patients categorized by mental health diagnoses at the psychiatric hospital and outpatient centers in Riga, Latvia, 2019.

Diagnosis (ICD-10 code)^a^ (10)	Number of patients (%)		
	Hospital^b^	First outpatient center	Second outpatient center
	Inpatients (*n* = 4,901)	Patients (*n* = 425)	Patients (*n* = 399)
Organic, including symptomatic, mental disorders (F0)	1,370 (28.0)	60 (14.1)	57 (14.3)
Schizophrenia, schizotypal, and delusional disorders (F2)	2,345 (47.8)	140 (32.9)	169 (42.4)
Mood (affective) disorders (F3)	634 (12.9)	185 (43.5)	159 (39.8)
Neurotic, stress-related, and somatoform disorders (F4)	346 (7.1)	38 (8.9)	13 (3.3)
Disorders of adult personality and behavior (F6)	51 (1.0)	2 (0.5)	1 (0.3)
Mental retardation (F7)	134 (2.7)	0 (0.0)	0 (0.0)
Behavioral and emotional disorders with onset usually occurring in childhood and adolescence (F8)	21 (0.4)	0 (0.0)	0 (0.0)

NA, not applicable; ICD 10, International Classification of Diseases.

Note that inconsistencies may arise due to rounding.

Source: Riga Psychiatry and Narcology Center.

^a^ The categories are based on the main diagnostic groups from the International Statistical Classification of Diseases and Related Health Problems, 10th edition.

^b^ The Riga Psychiatry and Narcology center is the only psychiatric hospital in Riga, Latvia.

**Table 2:** Percentage of patients categorized by mental health diagnoses at the psychiatric hospital before (2005) and after (2019) the opening of the outpatient centers in Riga.

Diagnosis (ICD-10 code)^a^	Number of patients (%)	
	Hospital^b^ (2005)	Hospital^b^ (2019)
	Inpatients (*n* = 5,491)	Inpatients (*n* = 4,901)
Organic, including symptomatic, mental disorders (F0)	1,365 (24.8)	1,370 (28.0)
Schizophrenia, schizotypal, and delusional disorders (F2)	2,992 (54.5)	2,345 (47.8)
Mood (affective) disorders (F3)	374 (6.8)	634 (12.9)
Neurotic, stress-related, and somatoform disorders (F4)	497 (9.0)	346 (7.1)
Disorders of adult personality and behavior (F6)	93 (1.7)	51 (1.0)
Mental retardation (F7)	170 (3.1)	134 (2.7)
Behavioral and emotional disorders with onset usually occurring in childhood and adolescence (F8)	0 (0.0)	21 (0.4)

NA, not applicable; ICD 10, International Classification of Diseases.

Note that inconsistencies may arise due to rounding.

Source: Riga Psychiatry and Narcology center.

^a^ The categories are based on the main diagnostic groups from the International Statistical Classification of Diseases and Related Health Problems, 10th edition.

^b^ The Riga Psychiatry and Narcology center is the only psychiatric hospital in Riga, Latvia.

Payment mechanisms for health services can affect service providers’ intentions to provide some services. The annual hospital budget in Latvia is calculated based on the number of treatment cases with minimum and maximum limits of hospitalizations (11). This could be a reason why the number of hospitalizations did not drop but was mostly stable. Outpatient visits are paid based on the number of episodes; day hospitals are paid based on the fee for services payment method. Outpatient service providers have an interest in providing more services.

The number and length of stay of patients treated at the inpatient hospital facility and the number of day patients have significantly decreased in Riga since 2020, which is likely associated with the COVID-19 pandemic. There was an extensive spread of COVID-19 in psychiatric hospitals, resulting in a limited capacity to admit patients (12). The work of the day clinic was also periodically suspended to limit the spread of COVID-19, and patients with milder COVID-19 (and a stable mental state) were discharged as early as possible and continued their treatment at home under the supervision of a family doctor (13). New patients were not admitted to the wards until all COVID-19 patients had recovered, were transferred to general hospitals, or discharged. These steps significantly reduced the number of patients treated at psychiatric hospitals.

## Actionable recommendations

Day clinics can, to a certain extent, reduce the length of stay in psychiatric hospitals. However, solutions for reducing the total number of patients with psychotic and organic psychiatric disorders treated in inpatient facilities may need to be sought elsewhere, such as through case management or home care. Day clinics could also target patients with specific psychiatric diagnoses, thereby restricting the admission of patients with other conditions to hospitals.

During epidemics or pandemics, it is expected that the treatment options for psychiatric patients will be limited, both in terms of the number of patients treated and the length of hospital stays. Outpatient care and day services would also be limited, without alternative care options being available. Therefore, it is necessary to be able to promptly restructure how work is conducted in psychiatric wards to reduce the possibility of patients infecting one another.

## Conclusion

In Riga, transforming psychiatric services from hospitals to outpatient clinics was possible and necessary. The outpatient centers included psychiatrist appointments, day clinics, and open-door inpatient wards, which were useful as an alternative to psychiatric hospitals. While the day clinics partially reduced the total length of stay in inpatient wards, they did not reduce the total number of patients treated. In Riga, the open-door inpatient wards (which offered more comprehensive treatment and rehabilitation services) increased the opportunity for patients with depression to receive treatment, which expanded the range of diagnoses of treated patients. However, this did not completely address the issues related to the quality of treatment of psychiatric hospital inpatients diagnosed with psychotic and/or organic psychiatric disorders. Moreover, the COVID-19 pandemic reduced opportunities for psychiatric patients to receive help. However, these conclusions refer to the situation in Riga and cannot be unequivocally applied to other regions or countries. Hence, further research investigating the influence of region-specific factors on providing treatment to psychiatric patients is necessary.
