# Supplementary material for: The potential of psychiatric outpatient centers to reduce the length of stay in inpatient facilities and the negative impact of COVID-19 on the availability of psychiatric services: the case of Latvia
Source: Front Health Serv. 2024 Jul 4;4:1348919. doi: 10.3389/frhs.2024.1348919 (PMC11256022; doi:10.3389/frhs.2024.1348919)
Supplement: Supplementary file 4 [file Table3.docx]

**TABLE 2** Percentage of patients by mental health diagnoses at the psychiatric hospital before (2005) and after (2019) the opening of outpatient centers, Riga

| **Diagnosis (ICD-10 code)^a^** | **Number of patients (%)** | |  |  |
| --- | --- | --- | --- | --- |
|  | **Hospital^b^ (2005)** | **Hospital^b^ (2019)** | |  |
|  | **inpatients (n=5491)** | **inpatients (n=4901)** | |  |
| Organic, including symptomatic, mental disorders (F0) | 1365 (24.8) | 1370 (28.0) | |  |
| Schizophrenia, schizotypal, and delusional disorders (F2) | 2992 (54.5) | 2345 (47.8) | |  |
| Mood (affective) disorders (F3) | 374 (6.8) | 634 (12.9) | |  |
| Neurotic, stress-related, and somatoform disorders (F4) | 497 (9.0) | 346 (7.1) | |  |
| Disorders of adult personality and behavior (F6) | 93 (1.7) | 51 (1.0) | |  |
| Mental retardation (F7) | 170 (3.1) | 134 (2.7) | |  |
| Behavioral and emotional disorders with onset usually occurring in childhood and adolescence (F8) | 0 (0.0) | 21 (0.4) | | |

NA: not applicable; ICD 10: International Classification of Diseases.

^a^The categories are based on the main diagnostic groups from the International Statistical Classification of Diseases and Related Health Problems, 10th edition.

^b^The Riga Psychiatry and Narcology center is the only psychiatric hospital in Riga, Latvia.

Note: Inconsistencies may arise due to rounding.

Source: Riga Psychiatry and Narcology center.
