# Supplementary material for: The potential of psychiatric outpatient centers to reduce the length of stay in inpatient facilities and the negative impact of COVID-19 on the availability of psychiatric services: the case of Latvia
Source: Front Health Serv. 2024 Jul 4;4:1348919. doi: 10.3389/frhs.2024.1348919 (PMC11256022; doi:10.3389/frhs.2024.1348919)

Figure 1. No of in-patients, no of day care patients, no of in-patient's days, 2004-2022 (blu - no of in-patients, red - no of treated day care patients, green - no of in-patient days (thousands))

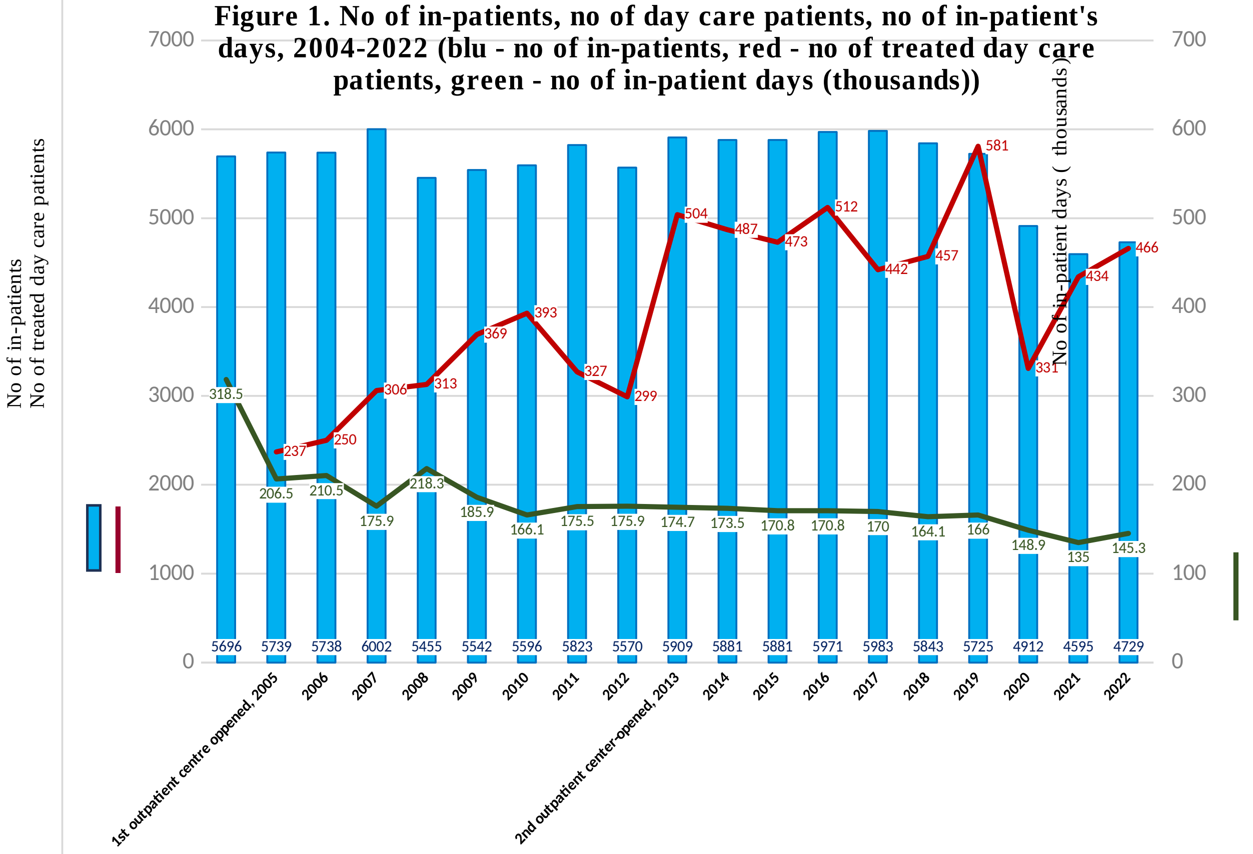

Supplement: Supplementary file 4 [file Table1.docx]
